# Effect of Polyethylene Glycol Content and Molar Mass on Injection Molding of Hydroxypropyl Methylcellulose Acetate Succinate-Based Gastroresistant Capsular Devices for Oral Drug Delivery

**DOI:** 10.3390/polym11030517

**Published:** 2019-03-19

**Authors:** Francesco Briatico-Vangosa, Alice Melocchi, Marco Uboldi, Andrea Gazzaniga, Lucia Zema, Alessandra Maroni

**Affiliations:** 1 Dipartimento di Chimica, Materiali e Ingegneria Chimica “G. Natta”, Politecnico di Milano, Piazza Leonardo da Vinci 32, 20133 Milan, Italy; francesco.briatico@polimi.it; 2 Dipartimento di Scienze Farmaceutiche, Sezione di Tecnologia e Legislazione Farmaceutiche “Maria Edvige Sangalli”, Università degli Studi di Milano, 20133 Milan, Italy; alice.melocchi@unimi.it (A.M.); marco.uboldi@unimi.it (M.U.); andrea.gazzaniga@unimi.it (A.G.); alessandra.maroni@unimi.it (A.M.)

**Keywords:** HPMCAS, PEG, plasticization, injection molding, drug delivery, gastric resistance, capsular device

## Abstract

Capsular devices for oral drug delivery were recently proposed and manufactured by injection molding (IM) as an evolution of traditional reservoir systems comprising a core and a functional coating. IM allowed the fabrication of capsule shells with release-controlling features based on the employed materials and the design characteristics. These features are independent of the drug, with significant savings in development time and costs. In previous work, IM was used to produce enteric-soluble capsules from blends of hydroxypropyl methylcellulose acetate succinate, with polyethylene glycol (PEG) as the plasticizer. In this work, the range of plasticizer concentrations and molar mass was broadened to evaluate in-depth how those parameters affect material processability and capsule performance over time. As expected, increasing the amount of the low molar mass plasticizer decreased the viscosity and modulus of the material. This simplified the molding process and enhanced the mechanical resistance of the shell, as observed during assembly. However, some samples turned out translucent, depending on several factors including storage conditions. This was attributed to plasticizer migration issues. Such results indicate that higher molar mass PEGs, while not significantly impacting on processability, lead to capsular devices with consistent performance in the investigated time lapse.

## 1. Introduction

Over the last few years, injection molding (IM) has started to be employed in the pharmaceutical field, primarily at the research level for testing its feasibility in the manufacturing of drug products [1]. The possibility of producing either immediate-release (IR) dosage forms or drug delivery systems (DDSs) has been evaluated. DDSs are able to control the rate, time and/or site of drug release, fulfilling therapeutic needs that could not be met by IR products [2,3,4,5,6,7,8]. The rising interest towards the use of IM in DDS manufacturing derives from the intellectual property associated with the resulting drug products, the flexibility in terms of design and composition, the reduction of both time and costs of production, and the possibility of avoiding the use of water as the solvent. Moreover, operating conditions, typically involving high pressure and temperature, could reduce microbial contamination on the one hand, and promote drug-polymer interactions on the other. This may lead to the formation of solid solutions increasing the dissolution rate of poorly soluble active ingredients. However, in order to avoid any degradation phenomena, processing temperatures need to be selected according to the thermal stability of the active molecules contained in the formulation.

Recently, IM has been proposed to manufacture functional capsular containers, i.e., systems with innovative design, composition and performance characteristics intended to be filled with the active ingredient after molding [9,10,11]. Such containers would be suitable for conveying various drug formulations (e.g., powders, pellets, non-aqueous liquid preparations) and also for modifying the release of the drug. In this respect, they represent an evolution of traditional reservoir DDSs based on solid dosage form cores (e.g., tablets, gelatin capsules, granules) coated with a release-controlling polymeric barrier [12]. The coating layer would be replaced by the molded functional capsule shell, composed of a cap and body manufactured separately to be assembled after filling. The performance of such capsules depends on the shell composition and design features (e.g., morphology and thickness), irrespective of the specific characteristics of the formulation contained in the DDS. This may enable the independent development of inner formulation and container shell, which would offer major advantages in terms of time and cost to market. Using hydroxypropyl cellulose (HPC), capsular devices able to impart a lag phase prior to the release of their contents (Chronocap™) were successfully manufactured by IM. The possibility of modulating the duration of the lag phase by varying the thickness of the shell and its composition, mainly the molar mass of the HPC, was also demonstrated. This pointed out suitability of the Chronocap™ oral delivery platform for chronotherapy and for targeting the colonic region based on a time-dependent approach [9,10,13,14,15,16]. Furthermore, the prototyping ability of 3D printing versus IM was proved when manufacturing Chronocap™. 3D printing could speed up the screening of formulation and design development stages by overcoming the need for multiple molds and the revision of the entire molding process [17,18]. The feasibility of IM in the manufacturing of enteric-soluble capsular devices was also investigated using hydroxypropyl methylcellulose acetate succinate (HPMCAS), soluble at pH ≥ 5.5, plasticized with polyethylene glycol (PEG) [19]. Such devices were intended to remain intact in the acidic environment of the stomach and to release their contents into the small bowel due to the dissolution of the shell at the higher pH values of the intestine. The obtained capsules were shown to possess suitable physico-technological characteristics, and the desired release performance.

To successfully apply IM in the manufacturing of drug products, the formulation step is especially challenging. It is essential to fulfill quality, efficacy and safety requirements for the final products. At the same time, the thermo-mechanical properties and rheological behavior of the components also have to be considered, in order to ensure proper moldability and stability over time. The research activities performed so far in the pharmaceutical field generally consist of feasibility studies. In these works, the main goals were the fabrication of prototypes by IM, and their characterization in terms of release performance. Only a few attempts to systematically evaluate the moldability of polymers approved for pharmaceutical use have been reported in the scientific literature [20,21]. In this respect, it was highlighted that the role of plasticizers is important to reduce the operating temperatures and decrease the risk of degradation. However, possible migration/leaching out of these additives has been widely reported in the case of food packaging and medical devices, and this problem has been associated with severe changes in the mechanical performance of end-products [22,23,24]. Notably, when dealing with drug products, such phenomena may affect not only the mechanical properties of the molded item but also its interaction with biological fluids, possibly affecting also its release behavior. A common strategy adopted in the plastics industry to limit migration of plasticizers and increase physical stability of molded products is the use of high molar mass plasticizing agents [25,26]. These agents are characterized by a lower diffusion rate in the polymer matrix, and are therefore more effectively retained in the molded product. However, their plasticizing efficiency may be reduced.

Based on these preliminary considerations, the aim of the present work is to systematically explore the effect of the addition of PEG plasticizers having different molar mass and concentration to HPMCAS, in order to investigate how these parameters affect the IM processability of the material and the performance of the resulting enteric-soluble capsular devices on an in-depth basis, also taking possible stability issues into account.

## 2. Materials and Methods

### 2.1. Materials

Hydroxypropyl methylcellulose acetate succinate (HPMCAS, cellulose, 2-hydroxypropyl methyl ether, acetate, hydrogen butanedioate; AQUOT-LG^®^, Shin-Etsu, Tokyo, Japan) was employed as the main component of the gastroresistant capsule shells in view of its solubility at pH ≥ 5.5, its suitability for hot-processing and the experience acquired on similar IM processes [19,27,28]; polyethylene glycol (PEG; Clariant Masterbatches, Milan, Italy) was chosen as the plasticizer based on previous processing use, and PEG 1500 (PEG1.5; 1400–1600 g/mol), PEG 8000 (PEG8.0; 7300–9000 g/mol) and PEG 20,000 (PEG20; 16,000–25,000 g/mol) were selected in order to evaluate the effect of the molar mass of the plasticizer. Acetaminophen (AAP, Rhodia, Milan, Italy) powder and the dye-containing formulation Kollicoat IR^®^ brilliant blue, based on a polyvinyl alcohol-polyethylene glycol graft-copolymer (BASF, Monza Brianza, Italy), were employed to fill the molded shells. AAP was then used to study the release performance of the DDSs, and the dye-containing formulations were used to visually check their opening.

HPMCAS was kept in an oven at 40 °C for 24 h prior to use. Plasticized polymeric formulations were prepared by mixing HPMCAS powder with the selected plasticizer in a mortar (Table 1). The amount of PEG was expressed as % by weight (wt %) on the dry polymer. In order to investigate the effect of the amount of plasticizer, PEG1.5 was mixed with HPMCAS in 15 wt %, 25 wt % and 35 wt% proportions, corresponding to a mass fraction, X_mass_, of 0.13, 0.2, and 0.26, respectively. The effect of the molar mass of the plasticizer was studied with the polymeric formulations containing 35 wt % of PEG, as this concentration was expected to provide the best processability.

Polymeric formulations were directly loaded into the IM press without any further processing, minimizing the exposure to high temperatures and shear stresses.

In order to produce samples for dynamic mechanical characterization, the same materials underwent hot melt extrusion in a counter-rotating twin-screw extruder (Haake™ MiniLab II microcompounder, Thermo Scientific, Milwaukee, WI, USA; screw diameter 5/14 and length 109.5 mm) with a rectangular cross-section die (thickness 1 mm, width 4 mm). The extrusion temperature was set at 170 °C for unplasticized HPMCAS powder and 160 °C for all plasticized polymeric formulations; the screw speed was 60 rpm. 50 mm long bars were obtained by cutting the extrudates immediately after production. Some samples were kept at ambient conditions, others were kept at 40 °C in an oven. Digital photographs (Nikon D70, Nikon, Tokyo, Japan) of the samples were also taken immediately after extrusion and at pre-determined time intervals.

### 2.2. Methods

#### 2.2.1. Rheological Characterization of Materials

The flow behavior of the materials was investigated by measuring the pressure drop between two sensors located at the entrance and exit of a slit capillary die (width 10 mm, height 1.5 mm, length 75 mm) integrated in the recirculation channel of the Haake™ MiniLab II microcompounder previously mentioned, equipped with two conical screws (in counter-rotating configuration). A protocol similar to that described by Casati F. et al. [21] was applied, and pressure drop and rotation speed data were processed as described by Yousfi et al. [29]. Tests were performed at temperatures between 170 °C and the minimum temperature allowed by the torque and pressure limitations of the extruder motor and pressure transducers, respectively. At least three tests were performed for each sample and temperature, and the average and standard deviation (sd) were calculated.

#### 2.2.2. Differential Scanning Calorimetry (DSC)

DSC analyses were performed by DSC Q100 (TA Instruments, New Castle, DE, USA; *n* = 1), using nitrogen as a purge gas (70 mL/min). An empty aluminum pan was used as the reference standard. Samples of about 5 mg were heated in aluminum crucibles from −50 to 240 °C, maintained at this temperature for 1 min, cooled down to −50 °C and reheated up to 240 °C. Both heating and cooling steps were run at 10 °C/min. DSC experiments were carried out on unplasticized HPMCAS, plasticized polymeric formulations and specimens cut from extrudates at different times.

#### 2.2.3. Dynamic Mechanical Analysis (DMA) of Extruded Samples

DMA was performed to assess the effect of the amount and molar mass of PEG on the mechanical behavior and glass transition of the considered material, and to investigate its stability over time and after thermal treatment. A TA RSA-III (TA Instruments, New Castle, DE, USA) dynamical mechanical analyzer was used to characterize samples extruded as described in the Materials section. Tests were performed immediately after extrusion and after 1, 3 and 10 days of treatment in an oven at *T*_treatment_ = 40 °C. Further, samples kept in air and at room temperature were considered, as discussed in the Results section.

The test protocol consisted in a temperature ramp of 3 °C/min from room temperature to the maximum temperature allowed by the sample before flowing. Tests were performed in tension, on bars with nominal gauge length, thickness and width of 20, 1.8 and 5 mm, respectively. To ensure linearity, measurements were carried out imposing a strain amplitude of 0.1% and at a 10 Hz frequency. To ensure that the sample was in tension throughout the whole test, a dynamic tracking algorithm allowed applying a static load 10% higher than the maximum dynamic load measured for any cycle.

**Figure 1:**
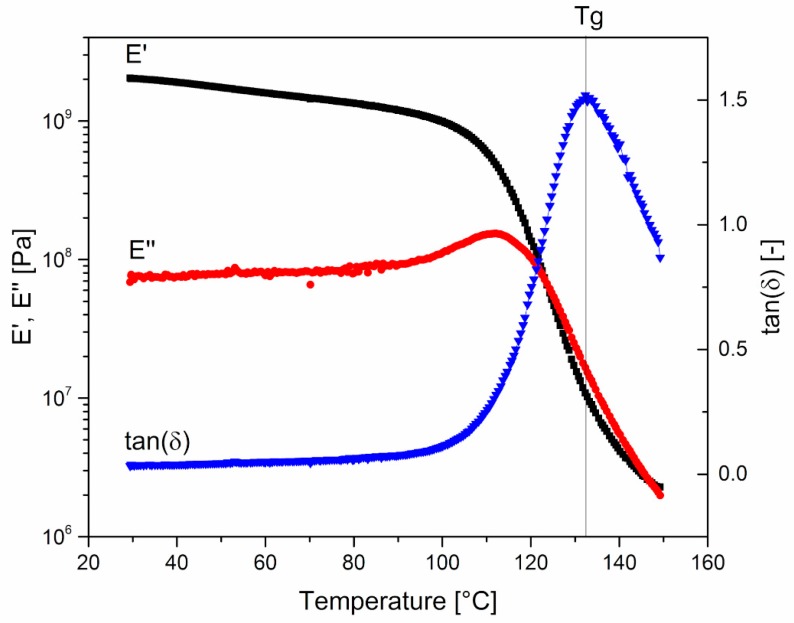
0-PEG. Temperature dependence of the conservative and dissipative components of the dynamic modulus, E’ and E’’, respectively, and of the loss factor, tan(δ) = E’’/E’. *T*_g_ is defined as the temperature at maximum of tan(δ) curve.

Figure 1 reports the temperature dependence of the conservative and dissipative components of the dynamic modulus, E’ and E’’ respectively, and of the loss factor, tan(δ) = E’’/E’. The glass transition temperature (*T*_g_) is defined as the temperature at which tan(δ) reaches its maximum value.

#### 2.2.4. IM of Capsular Devices

IM processes were performed using a benchtop micro-molding press (BabyPlast 6/10P, Cronoplast S.L., Rambaldi S.r.l., Lecco, Italy) equipped with a mold composed of two interchangeable inserts for the manufacturing of the body and cap of capsular devices with constant nominal thickness of 600 µm [10]. The mold was characterized by the presence of a single cavity and entailed (i) a hot runner system to maintain the desired temperature during mold filling, preventing overheating in the upstream components of the IM machine, (ii) a length/diameter ratio of 1.5 to limit the flow path, (iii) a centered injection position to enable a balanced flow of the material in all directions, and (iv) a duct for injection of compressed air into the mold to ease ejection of the molded part. Each capsular device was composed by two parts—a body and a cap. The thickness of the wall of both parts was halved in the area where they overlapped to lock the device. This way, wall thickness was kept constant across the whole assembled device.

Polymeric formulations were loaded into the IM press through a hopper. An amount of material (C) defined by the final position of the injecting plunger (ø = 10 mm) was then forced into a plasticating chamber containing heated spheres. The molten material then accumulated in the injection chamber. Both the injection and holding phases were performed in pressure control, with the injection pressure (P_1_) maintained for 2.5 s and the packing pressure (P_2_) maintained for 1.5 s. The constant pressure value was reached by moving the injection piston at selected rates (r_1_ and r_2_ for injection and holding, respectively); such rates are expressed as a percentage of the maximum rate achievable by the machine. The diameter of the injection nozzle was 1 mm. Four different temperatures (T_1_–T_4_) were set throughout the equipment, where the last one was the temperature of the hot runner of the molding system. The IM operating conditions for each material are reported in Table 2.

#### 2.2.5. Characterization of Capsular Devices

**Figure 2:**
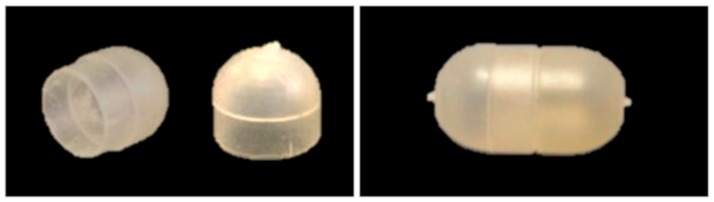
Photographs of 25-PEG1.5-based body and cap (**left**) and assembled capsular device (**right**).

Bodies and caps were checked for weight (analytical balance BP211, Sartorius, Göttingen, Germany; *n* = 10) and thickness (MiniTest FH7200 equipped with FH4 probe, ø sphere = 1.5 mm, ElektroPhysik, Köln, Germany; *n* = 10). Digital photographs (Nikon D70, Nikon, Tokyo, Japan) were also taken (Figure 2).

**Figure 3:**
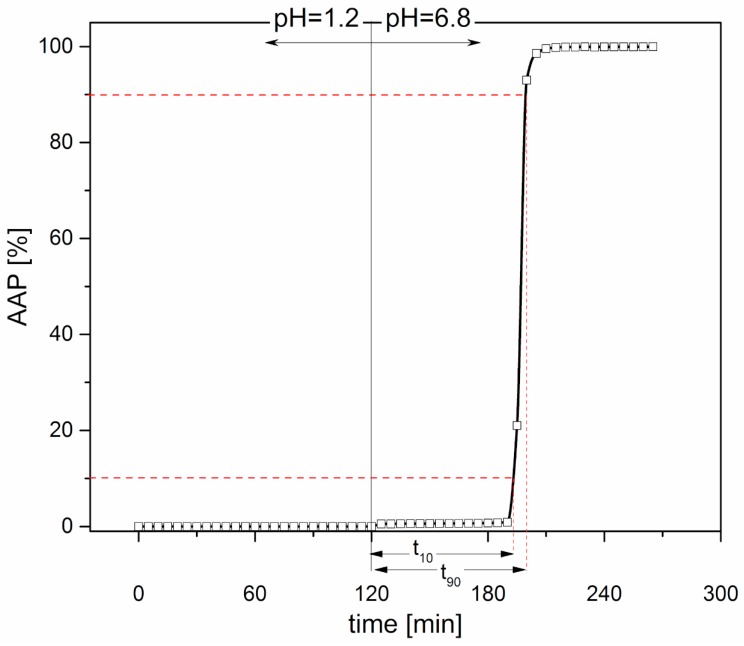
Release profile of a 25-PEG1.5-based capsular device; calculated t_10%_ and t_90%_ − t_10%_ were about 72 min and 7 min, respectively.

Release performance-Capsule bodies were manually filled with approximately 80 mg of AAP (CV ≤ 2%) and then closed with matching caps. The compendial test for gastroresistant dosage forms (Dissolution Test for Delayed-Release Dosage Forms-Method B, USP38) was performed in apparatus 2 (Dissolution System 2100B, Distek, North Brunswick Township, NJ, USA; *n* = 3) at 100 rpm and 37 ± 0.5 °C. The devices were maintained in hydrochloric acid buffer pH 1.2 for 2 h, to simulate the gastric environment, and then transferred into phosphate buffer pH 6.8, to mimic the pH change after stomach emptying. Gastroresistant dosage forms are required to remain intact in the former stage of the test and release their drug content during the latter. Fluid samples were automatically withdrawn by a peristaltic pump every 5 min and the amount of drug released was assayed spectrophotometrically (cuvette with 1 mm optical path length; λ = 245 nm). Capsules were able to withstand the acidic medium (pH 1.2) for 2 h, whereas in the pH 6.8 buffer, there was a lag time prior to the break-up and release of the tracer. These results were in agreement with the behavior of traditional gastroresistant reservoir systems obtained by film coating and characterized by release-controlling layers of hundred microns in thickness [30,31]. The in vitro lag time was expressed as the time required for 10% drug release in phosphate buffer (t_10%_). It was calculated by subtracting 120 min (i.e., the time during which the devices were kept in pH 1.2 fluid) from the time obtained by linear interpolation of the experimental data immediately before and after this release percentage. By analogously calculating t_90%_ and subtracting t_10%_ from the resulting value, an index of the time needed for the complete release of the tracer was obtained (t_90%_ − t_10%_). By way of example, the release profile of a 25-PEG1.5-based capsular device is reported in Figure 3.

Opening behavior—To investigate the opening mechanism of capsular devices, capsule body parts were manually filled with Kollicoat IR^®^ brilliant blue and then closed with the matching caps. The filled capsules were immersed in unstirred pH 6.8 buffer at room temperature and digital photographs were taken at successive time points.

## 3. Results and Discussion

### 3.1. Characterization of HPMCAS-Based Materials

The rheological investigation was performed in order to assess the processability of HPMCAS, in a process temperature window limited by the need to avoid the degradation of the material that would impair its application to the manufacturing of drug products. During the development of industrial processes, it is necessary to assess the stability of the drug/materials and of the product, in order to rule out the formation of any hazardous degradation products and to ensure quality and safety [18].

**Figure 4:**
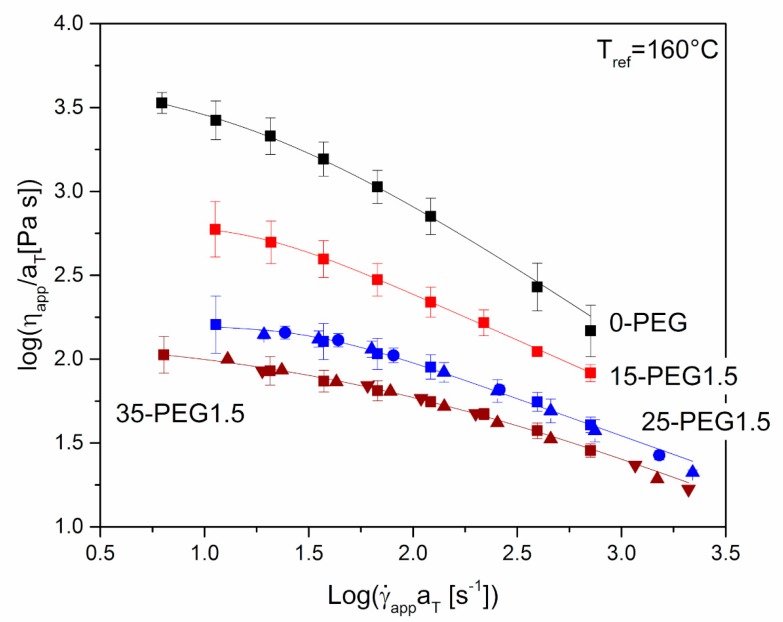
Steady-state apparent shear viscosity, η_app_, versus apparent shear rate at die wall, $\dot{\gamma }$_app,w_, for 0-PEG, 15-PEG1.5, 25-PEG1.5, 35-PEG1.5 from capillary rheometry tests at: 160 °C (■), 155 °C (●), 150 °C (▲), 140 °C (▼). The reference temperature for 25-PEG1.5 and 35-PEG1.5 master curves is 160 °C. Lines represent the fitting with Cross equation (Equation (2)).

Figure 4 shows the dependence of apparent shear viscosity on apparent shear rate for either unplasticized HPMCAS or HPMCAS containing different amounts of PEG1.5 as the plasticizer. In the case of unplasticized HPMCAS and 15-PEG1.5, it was possible to perform the test at 160 °C only, because at lower temperatures the maximum torque of the extruder was exceeded. For formulations containing higher amounts of plasticizer, it was possible to carry out the experiment at lower temperatures, and a master curve was built. The relevant shift factors, a_T_, showed an Arrhenius type dependence, Equation (1):

(1) $$a_{T}\left(T\right)=exp\left(\frac{E_{0}}{R}\left(\frac{1}{T}-\frac{1}{T_{ref}}\right)\right)$$

where *T_ref_* is the reference temperature, *R* the gas constant and *E*_0_ the viscous flow activation energy, ≈184 kJ/mol for 25-PEG1.5 and ≈ 87 kJ/mol for 35-PEG1.5. The lower value of the activation energy in the second case may be attributed to the greater plasticization effect of the higher amount of PEG. In general, the values of *E*_0_ were significantly higher than those of common thermoplastic polymers, which are usually limited to few tens of kJ/mol and are always lower than 100 kJ/mol [32,33]. However, even higher values were observed for other cellulosic polymers by Baldi et al. [20], who explained their observation either with the presence of partially unmolten material or with partial degradation of the material during rather long tests at high temperature under shear. The same explanation may probably be adopted in the present case.

Figure 4 reports also the fitting of data by Cross equation, Equation (2):

(2) $$\frac{\eta -\eta_{\infty }}{\eta_{0}-\eta_{\infty }}=\frac{1}{1+{\left(\lambda \dot{\gamma }\right)}^{m}}$$

where *λ* is a time constant, m the power law index and *η*_0_ and *η*_∞_ the limiting viscosities at zero and infinite shear rates. The resulting fitting parameters are reported in Table 3. When computing the fitting parameters, *η*_∞_ was set equal to 0.

By comparing both the curves in Figure 4 and the zero shear viscosity, *η*_0_, in Table 3, the plasticizing effect of PEG is evident, as increasing the amount of plasticizer markedly reduces the viscosity, making the polymer easier to process. Therefore, the presence of a higher amount of the plasticizer should favor the processing of the material, as also confirmed by the torque needed to extrude the materials from the microcompounder, equal to 150, 110, 90, 40 N cm for 0-PEG, 15-PEG1.5, 25-PEG1.5 and 35-PEG1.5, respectively.

**Figure 5:**
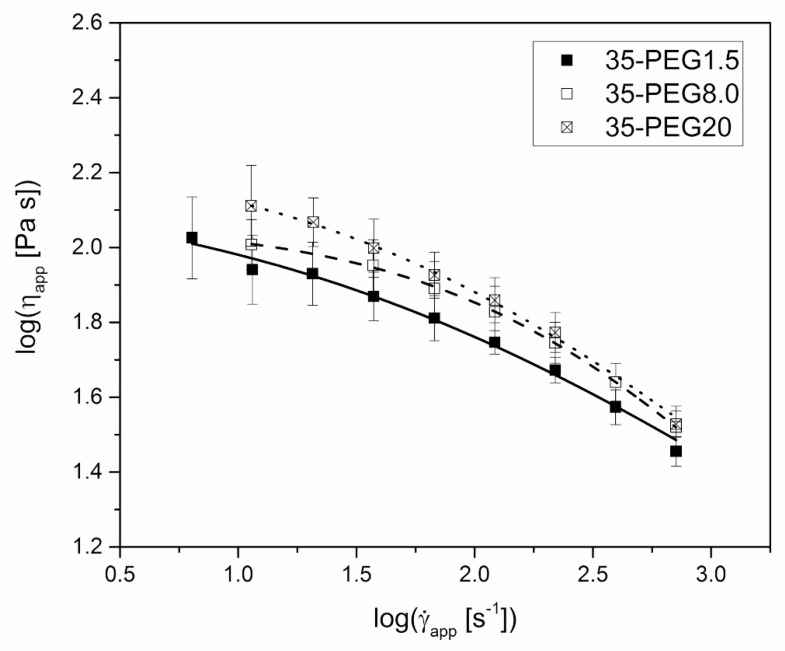
Steady-state apparent shear viscosity, η_app_, versus apparent shear rate at die wall, ${\dot{\gamma }}_{\mathrm{app}}$, at 160 °C for 35-PEG1.5, 35-PEG8.0 and 35-PEG20. Lines represent the fitting with Cross equation (Equation (2)).

The increase in the molar mass of PEG caused an increase in the apparent viscosity of formulations containing 35% of plasticizers, as reported in Figure 5. However, the impact of this parameter was limited if compared to that of the concentration of PEG at constant molar mass, and was expected to have a minor effect on the material processability.

**Figure 6:**
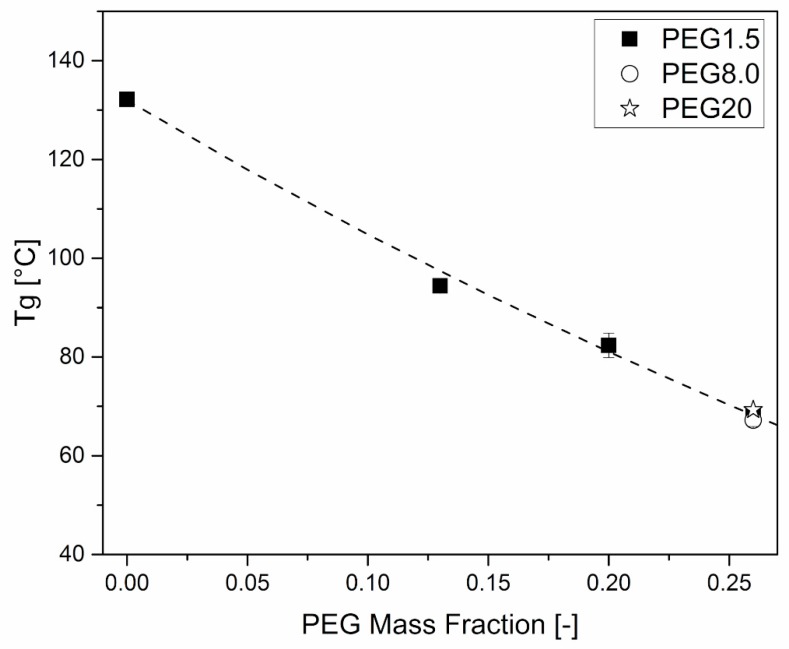
*T*_g_ dependence on PEG mass fraction of HPMCAS-based formulations. Tests were performed immediately after sample production. The interpolation line uses the Fox equation (Equation (3)).

**Figure 7:**
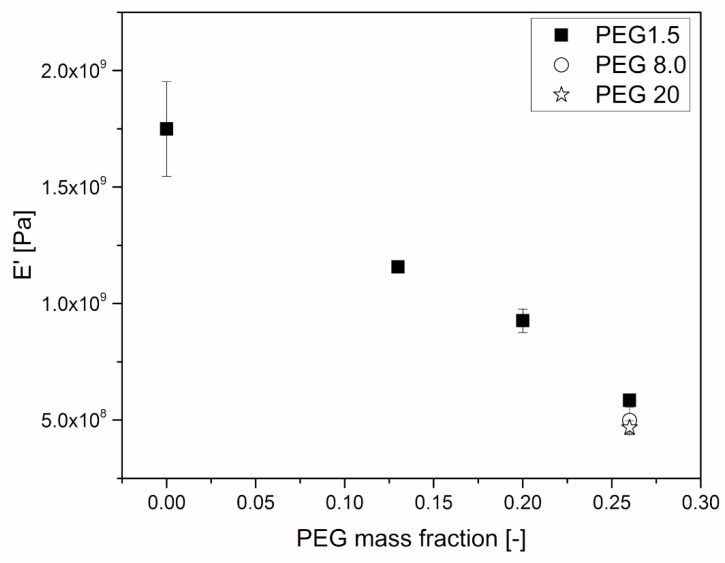
E’ dependence on PEG mass fraction of HPMCAS-PEG blends. Tests were performed immediately after sample production. The value was measured at 40 °C, during a temperature ramp experiment.

The effects of plasticizers on the mechanical behavior of HPMCAS were further investigated through DMA. Figure 6 and Figure 7 clearly show that the amount of plasticizer reduced the *T*_g_ and the modulus of the material at room temperature, respectively.

The dependence of *T*_g_ on temperature could be effectively fitted using the Fox equation, Equation (3):

(3) $$\frac{1}{T_{g}}=\frac{\chi_{1}}{T_{g1}}+\frac{1-\chi_{1}}{T_{g2}}$$

where χ_1_ is the PEG mass fraction, *T*_g2_ is the unplasticized HPMCAS glass transition and *T*_g1_ is the PEG glass transition. As the latter property was not available from experiments, it was left as a free fitting parameter, which had the value of ≅ −38 °C, consistent with the value of −42 °C determined by DSC for the same material by Bochmann et al. [34].

The conservative component of the complex modulus (E’) seems to depend linearly on PEG weight fraction, at least in the considered range of compositions. The dependence is however stronger than could be expected from a simple rule of mixtures, and this is probably also due to the reduction of *T*_g_ induced by PEG.

As for the effect of the molar mass of PEG, Figure 6 and Figure 7 show that it is limited, as in the case of viscosity.

**Figure 8:**
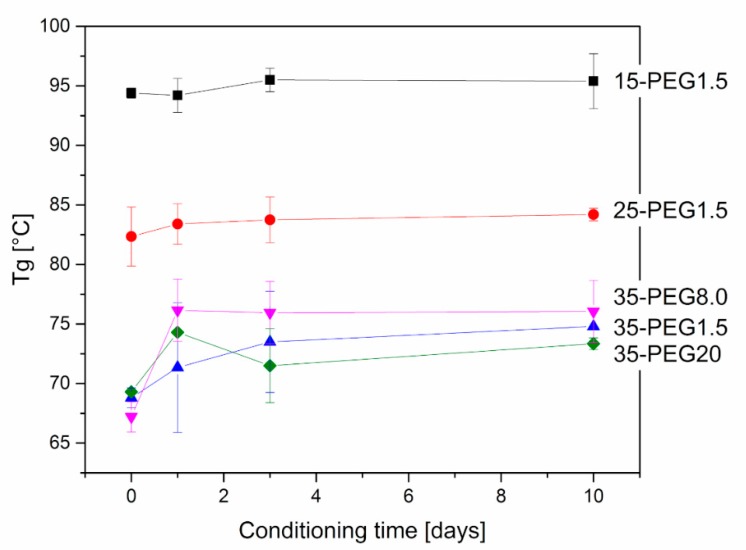
Variation in *T*_g_ of plasticized HPMCAS samples after thermal treatment at 40 °C. Effect of concentration of PEG1.5 and of molar mass in the case of 35-PEG.

**Figure 9:**
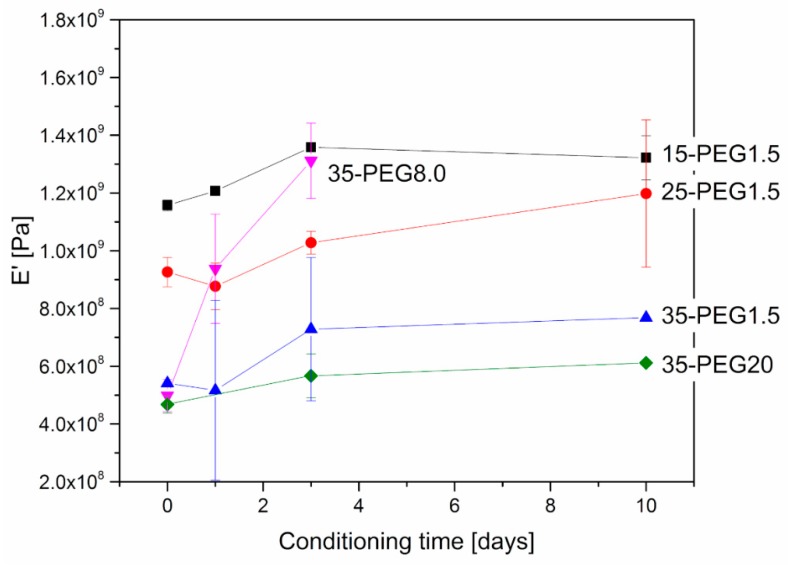
Variation in E’ of plasticized HPMCAS samples after thermal treatment at 40 °C. Effect of concentration of PEG1.5 and of molar mass in the case of 35-PEG.

Further, DMA allowed investigation of the stability of material properties over time. As reported in Figure 8 and Figure 9, at increasing conditioning times at 40 °C, both *T*_g_ and the moduli increased, which can be related to the release of PEG from HPMCAS. This is particularly evident at higher PEG concentrations. The effect of molar mass is in this case marked: the increase in both *T*_g_ and E’ was higher for PEG8.0 and PEG1.5, and more limited for PEG20.

### 3.2. Manufacturing and Characterization of HPMCAS-Based Capsular Devices

**Figure 10:**
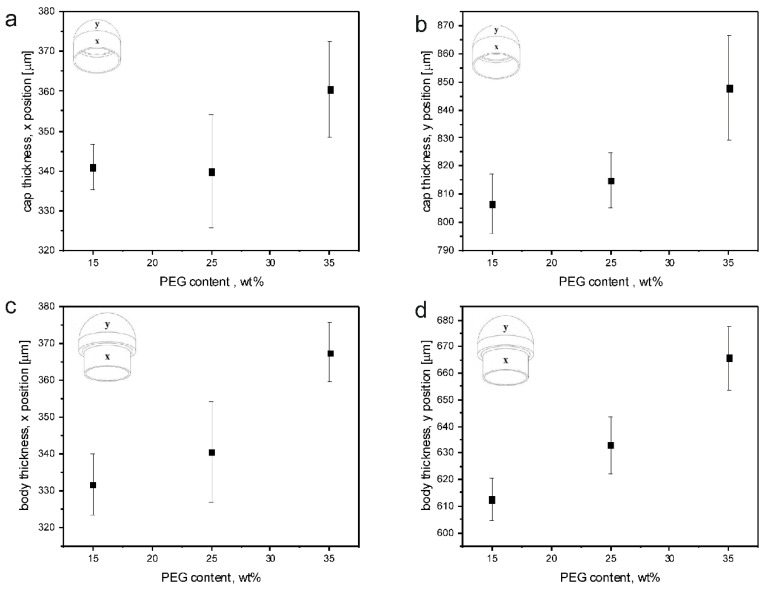
Average thickness values, with relevant sd, resulting from measurements performed on thin and thick sections of caps (**a**,**b**) and bodies (**c**,**d**) obtained from15-PEG1.5, 25-PEG1.5 and 35-PEG1.5 formulations.

IM of capsule bodies and caps was successfully performed with all the formulations investigated, except for unplasticized HPMCAS (0-PEG). Common operating conditions (i.e., suitable to mold all the plasticized formulations) were employed in order to allow a better comparison of the capsular devices. The items obtained, appearing transparent, were characterized for weight and shell thickness. Figure 10 shows the thickness data measured from the thin and thick sections of PEG1.5-based caps and bodies. The nominal thickness values are 300 µm (thin sections) and 600 µm (thick sections). Table 4 reports the weight data for the same formulations.

The weight data showed good reproducibility with all the tested formulations. On the other hand, the mismatch of measured versus nominal thickness values may be attributed to an overpacking during the holding phase of the IM process. This phenomenon might also explain the increase in thickness observed when the PEG content increases. The overpacking-related expansion is due to the difference in specific volume of the materials at the packing pressure at glass transition and at ejection conditions (typically room temperature and atmospheric pressure). The expansion increased with the absolute value of this difference. Since packing pressure was constant for all the molding experiments, and assuming that both the thermal expansion coefficient and the specific volume at ejection were independent of PEG concentration, the only difference among the various formulations could be related to the effect of PEG concentration on *T*_g_ that was previously reported. As PEG mass fraction increased, *T*_g_ was reduced: consequently, specific volume at glass transition decreased and the difference with specific volume at ejection increased. This caused a larger expansion of the capsule walls.

Capsule shells based on 15-PEG1.5 turned out to be too brittle, which was observed when filling them manually. Indeed, these capsules could not effectively be assembled without damaging the caps or breaking them up. Unplasticized HPMCAS and its 15% plasticized formulation were therefore discarded due to either unfeasible processing or poor mechanical resistance of the molded parts.

**Figure 11:**
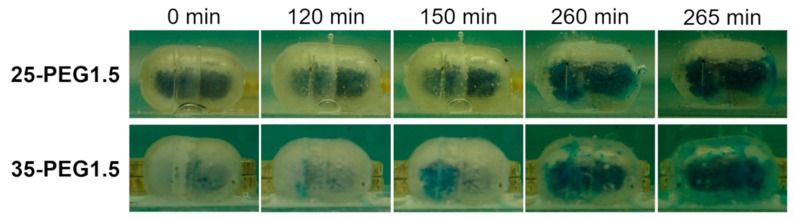
25-PEG1.5 and 35-PEG1.5-based capsular devices filled with a dye-containing formulation at different time points during immersion in unstirred pH 6.8 buffer. Break-up was highlighted by the presence of blue coloration outside the shell after 260 min.

All the capsular devices tested for interaction with aqueous fluids were able to resist in acidic medium irrespective of the amount and molar mass of the PEG employed and of the hydrodynamic conditions. In pH 6.8, the capsules were able to release their contents both in unstirred and stirred conditions (Figure 11, Table 5). Release occurred following dissolution of the polymeric barrier and final break-up of the shells, which took place some time after the pH change (lag time). The lag time was shown to be largely reduced under hydrodynamic conditions (≈60 min versus 4 h), as expected. Interestingly, it did not seem to be influenced by the type of plasticizer in the polymeric formulation, nor by its amount. Moving from 25% to 35% of PEG, despite the previously noted reduction in *T*_g,_ associated with an increase in HPMCAS free volume, no significant variation of t_10%_ was observed. In the same way, the addition of PEG with higher molar mass to HPMCAS, which was already demonstrated to have a minor effect on the *T*_g_, did not modify the release performance of the resulting capsular devices. Because of the lack of major changes in t_10%_, it could be hypothesized that an increased amount of plasticizer would make the dissolution and breaking up of the polymeric shell faster. This would counteract the effect of an increase in capsule thickness on the overall release performance.

Caps and bodies were stored at 40 °C for 30 days and then filled and assembled to evaluate the release performance of the capsular device over time. Samples based on the 35-PEG1.5 formulation lost their initial transparency and appeared translucent. This might be associated with the segregation of the plasticizer and its migration to the capsule surface. This phenomenon was accompanied by an increase of both the lag time and the release duration (t_10%_ and t_90%_ − t_10%_ in Table 5). PEG migration in the capsule was in agreement with the DMA evidence of moduli and tan(δ) shift for samples prepared by hot melt extrusion and then conditioned in an oven at 40 °C, which pointed out the possible leaching of PEG from the polymeric barrier. The increase in lag time may be partly related to the same migration effect, which may have an impact on the release performance of the capsular devices by leading to the presence of PEG-rich and PEG-depleted regions at the surface and in the bulk of the capsule wall, respectively. This would ultimately result in an overall reduction of the rate of water penetration into the polymeric barrier, also in turn reducing the dissolution rate. In this case, the polymeric network at the sample surface might be characterized by a higher concentration of hydrophilic/soluble plasticizer, improving water penetration rate, solvation of the polymer chains and final dissolution. However, the opposite situation should occur in the internal portion of the shell walls.

### 3.3. Characterization of Translucent HPMCAS-Based Samples

A thermal and mechanical analysis was carried out in order to investigate the change in transparency of the capsular devices in time. The aim was verifying possible PEG segregation/migration and checking to which extent PEG molar mass may affect it. Due to the limited number of available capsular devices and the impossibility of retrieving samples suitable for mechanical testing out of them, the investigation was carried out on extruded specimens, which after extrusion were stored either at ambient conditions or in an oven at 40 °C.

Visual inspection allowed to detect changes in the appearance both of samples stored at room temperature and at 40 °C. Interestingly, samples stored at room condition started getting translucent before than the samples stored at 40 °C. Furthermore, samples with different degrees of plasticization turned translucent at different times, in the 15-PEG1.5 ≈ 35-PEG8.0 > 25-PEG1.5 > 35-PEG20 > 35-PEG1.5 order.

**Figure 12:**
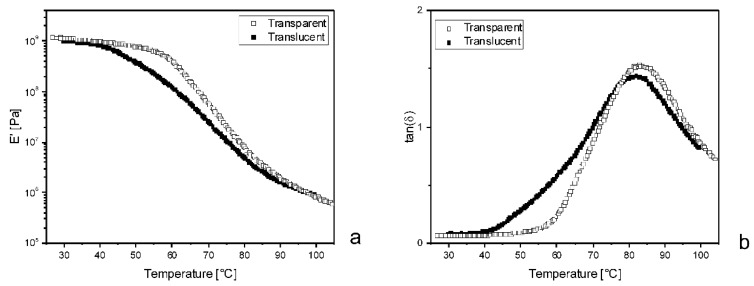
Comparison between the dynamical mechanical responses of 25-PEG1.5 either maintained in an oven at 40 °C, indicated as “Transparent”, or at room temperature, indicated as “Translucent”. (**a**) Conservative component of the complex modulus, E’, versus temperature; (**b**) Loss factor, tan(δ) versus temperature.

Although no systematic study was carried out, DMA performed on translucent samples highlighted remarkable differences with respect to transparent ones. As an example, Figure 12 shows the results from 25-PEG1.5, after 50 days in an oven or at room temperature.

Furthermore, it was observed that only a thin external layer of the samples aged in air was translucent, while the core was still transparent. This suggested that the appearance of the samples would be due to PEG concentration at their surface. The oven treatment probably favors a homogeneous release of PEG and may be considered as a beneficial post-processing treatment for the DDS.

**Figure 13:**
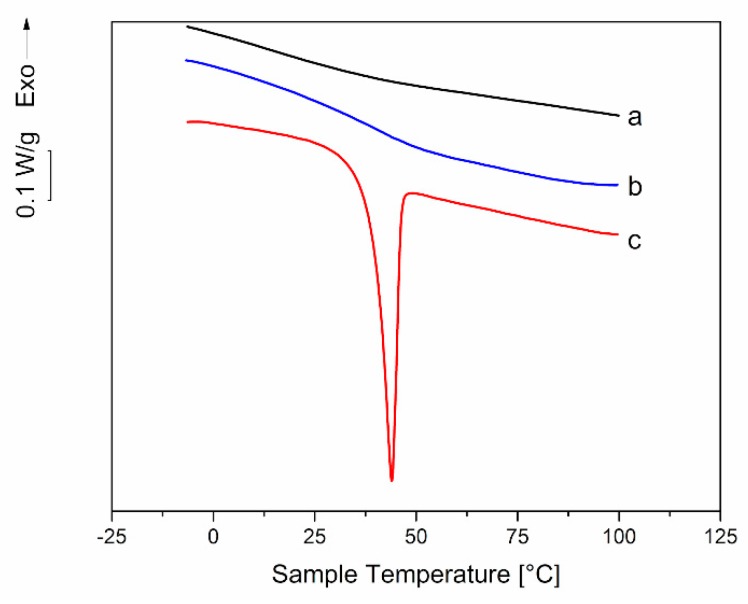
Comparison between the DSC thermograms of dynamical mechanical responses of (**a**) a 35-PEG1.5 extruded bar immediately after extrusion; (**b**) the inner, transparent region of a 35-PEG1.5 bar maintained at room temperature for 2 months after extrusion; (**c**) the external thin opaque layer of a 35-PEG1.5 bar maintained at room temperature for 2 months after extrusion.

To verify this hypothesis, DSC was employed to analyze material collected from aged samples and from completely transparent samples immediately after extrusion. In the first case, material was collected from both the thin external layer and the core. Figure 13 shows the thermogram for 35-PEG1.5: a deep melting endotherm with a melting temperature, *T*_m_, close to that of PEG 1500 (51 °C) can be observed only for the thin external layer. This seems to confirm the migration of PEG to the sample surface and its segregation. Similar endotherms were observed also for 25-PEG1.5, 35-PEG8.0 and 35-PEG20, with *T*_m_ close to the ones relevant to pure PEG (64 °C and 66 °C, respectively). However, in these cases the endothermic peaks were very small.

From the specific melting enthalpies, ΔH_melting_, measured in DSC for the layers and the pure PEGs, the PEG mass fraction, χ_PEG_, on the external layer could be estimated as (Equation (4)):

(4) $$\chi_{PEG}=\frac{\Delta H_{melting,\;thin\;layer}}{\Delta H_{melting,\;PEG}}$$

in which Δ*H_melting, thin layer_* and Δ*H_melting, PEG_* are the specific melting enthalpies for the thin opaque layer taken form the sample and pure PEG.

The results in Table 6 show that in the case of PEG1.5, a remarkable amount of PEG migrated to the surface. The amount is higher for HPMCAS loaded with the higher amount of PEG, suggesting a faster phenomenon in that case. On the other hand, migration at the same aging time was very limited in the case of 35-PEG8.0 and 35-PEG20: this would confirm the better suitability of higher molar mass PEGs as plasticizers for HPMCAS. However, a limited though still detectable migration was found even for the higher molar masses of PEG. This indicates that a longer investigation into stability should also be carried out at lower PEG concentrations.

## 4. Conclusions

Capsular devices suitable for filling after production with various drug-containing formulations to modify their release were recently described as an advantageous alternative to traditional coated reservoir systems. In this study, the feasibility of IM for manufacturing ready-to-use gastroresistant capsules was demonstrated starting from enteric-soluble HPMCAS. Considering the major role played by the plasticizer in the molding process and its impact on the mechanical as well as functional characteristics of the molded capsules, a systematic study was performed to evaluate the outcome of the addition to HPMCAS of PEGs of different molar mass and in differing concentrations.

The results obtained from rheological characterization showed that an increase in the amount of PEG markedly reduced the *T*_g_ of HPMCAS, and consequently reduced the viscosity of plasticized formulations. This made the IM process easier to carry out. The use of PEGs with higher molar mass was associated with a slightly increased apparent viscosity of the melt, but it did not result in major effects in terms of material processability. It was possible to successfully mold capsule bodies and caps with all the formulations investigated, plasticized with PEG 1500, 8000 and 20,000 in a 15–35 wt % concentration range. Items containing the lowest amount of PEG 1500 turned out to be too brittle to undergo the manual filling and assembly steps. On the other hand, the higher molar mass PEGs led to capsular devices with suitable physico-technological characteristics and consistent performance.

A progressive loss of transparency was observed in the samples after a range of time periods, which was also affected by the storage conditions. This could be related to a migration of PEG within the polymer matrix over time, as supported by DSC analyses. Such a phenomenon, leading to concentration of plasticizer in the outer region of the molded products, was found to be especially evident in the presence of the lowest molar mass PEG at the highest concentration. These results were consistent with previous literature data relevant to applications of IM to areas other than the pharmaceutical one, e.g., for packaging purposes, where its use is more consolidated. The leaching of PEG, appearing faster on storage at 40 °C, was associated with an increase in *T*_g_. However, it was not reflected in an alteration of the gastric resistance of the capsules.

Although possible PEG migration issues did not seem to raise major concerns in the specific experimental settings here investigated, the data collected may recommend a curing treatment. This treatment, performed after molding the capsule bodies and caps, would be beneficial to improve the physical stability of ready-to-fill enteric-soluble capsular devices based on HPMCAS.

## Figures and Tables

**Table 1:** Composition of the formulations investigated and relevant codes.

	PEG		Formulation Code
	Nominal Molar Mass	wt %	
**HPMCAS**	-	0	0-PEG
	1500	15	15-PEG1.5
		25	25-PEG1.5
		35	35-PEG1.5
	8000	35	35-PEG8.0
	20000	35	35-PEG20

**Table 2:** IM operating conditions.

Formulation	T_1_ (°C)	T_2_ (°C)	T_3_ (°C)	T_4_ (°C)	C (mm)	P_1_ (bar)	r_1_ (%)	P_2_ (bar)	r_2_ (%)
15-PEG1.5	130	135	155	165	6	30	0.4	20	0.3
25-PEG1.5	130	135	150	160	6	30	0.4	20	0.3
35-PEG1.5	130	135	150	160	7	30	0.4	20	0.3
35-PEG8.0	130	135	160	170	6	30	0.4	20	0.3
35-PEG20	130	135	160	170	6	30	0.4	20	0.3

**Table 3:** Fitting parameters of the Cross equation (Equation (2)) for unplasticized HPMCAS and its blends with different PEGs.

Formulation	*η*_0_ (Pa s)	*λ* (s)	m (–)	R^2^
0-PEG	5037 ± 389	0.071 ± 0.014	0.84 ± 0.04	0.998
15-PEG1.5	1003 ± 55	0.050 ± 0.008	0.68 ± 0.01	0.994
25-PEG1.5	236 ± 9	0.021 ± 0.003	0.55 ± 0.04	0.996
35-PEG1.5	141 ± 17	0.019 ± 0.004	0.52 ± 0.02	0.995
35-PEG8.0	115 ± 2	0.005 ± 0.003	0.71 ± 0.02	0.999
35-PEG20	184 ± 14	0.019 ± 0.005	0.55 ± 0.04	0.998

**Table 4:** Average weight values, with relevant sd, resulting from measurements performed on caps and bodies obtained from 15-PEG1.5, 25-PEG1.5 and 35-PEG1.5 formulations.

Formulation	Weight (mg)	
	Cap	Body
15-PEG1.5	114.57 (1.02)	125.79 (1.99)
25-PEG1.5	115.46 (0.62)	128.07 (0.28)
35-PEG1.5	115.85 (0.31)	127.51 (0.32)

**Table 5:** t_10%_ and t_90%_ − t_10%_ (sd in brackets) relevant to capsular devices of different composition immediately after molding and after 30 days storage at 40 °C.

Formulation	t_0_ (min)		t_30days_ (min)	
	t_10%_	t_90%_ − t_10%_	t_10%_	t_90%_ − t_10%_
25-PEG1.5	65.2 (6.1)	19.7 (11.9)	74.7 (6.4)	17.9 (8.1)
35-PEG1.5	60.3 (5.4)	13.6 (8.4)	78.0 (4.3)	82.9 (15.4)
35-PEG8.0	57.4 (7.1)	15.9 (6.1)	63.8 (5.7)	18.3 (8.4)
35-PEG20	56.7 (15.1)	20.5 (3.0)	68.6 (24.6)	14.52 (4.8)

**Table 6:** Melting enthalpies and estimated PEG mass fraction in the thin opaque layers of 25-PEG1.5, 35-PEG1.5, 35-PEG8.0 and 35-PEG20 bars maintained at room temperature for 2 months after extrusion. Δ*H_melting, PEG_* is 146, 158 and 159 J/g for PEG1.5, PEG8.0 and PEG20, respectively.

Formulation	Δ*H_melting, thin layer_* (J/g)	*χ*_*PEG*_ (%)
25-PEG1.5	5.0	3.4
35-PEG1.5	22.4	15.2
35-PEG8.0	0.3	0.3
35-PEG20	0.3	0.3
